# Protocol for the development of a repository of individual participant data from randomised controlled trials conducted in adult care homes (the Virtual International Care Homes Trials Archive (VICHTA))

**DOI:** 10.1186/s13063-021-05107-w

**Published:** 2021-02-23

**Authors:** Lisa Irvine, Jennifer Kirsty Burton, Myzoon Ali, Terence J. Quinn, Claire Goodman

**Affiliations:** 1 Centre for Research in Public Health and Community Care, University of Hertfordshire, Hatfield, UK; 2 Institute of Cardiovascular and Medical Sciences, University of Glasgow, Glasgow, UK; 3 NIHR Applied Research Collaboration East of England, Cambridge, UK

**Keywords:** Care homes, Long-term care, Individual participant data (IPD), Randomised trials, Minimum dataset, Data sharing

## Abstract

**Background:**

Approximately 418,000 people live in care homes in the UK, yet accessible, robust data on care home populations and organisation are lacking. This hampers our ability to plan, allocate resources or prevent risk. Large randomised controlled trials (RCTs) conducted in care homes offer a potential solution. The value of detailed data on residents’ demographics, outcomes and contextual information captured in RCTs has yet to be fully realised. Irrespective of the intervention tested, much of the trial data collected overlaps in terms of structured assessments and descriptive information. Given the time and costs required to prospectively collect data in these populations, pooling anonymised RCT data into a structured repository offers benefit; secondary analyses of pooled RCT data can improve understanding of this under-researched population and enhance the future trial design. This protocol describes the creation of a project-specific repository of individual participant data (IPD) from trials conducted in care homes and subsequent expansion into a legacy dataset for wider use, to address the need for accurate, high-quality IPD on this vulnerable population.

**Methods:**

Informed by scoping of relevant literature, the principal investigators of RCTs conducted in adult care homes in the UK since 2010 will be invited to contribute trial IPD. Contributing trialists will form a Steering Committee who will oversee data sharing and remain gatekeepers of their own trial’s data. IPD will be cleaned and standardised in consultation with the Steering Committee for accuracy. Planned analyses include a comparison of pooled IPD with point estimates from administrative sources, to assess generalisability of RCT data to the wider care home population. We will also identify key resident characteristics and outcomes from within the trial repository, which will inform the development of a national minimum dataset for care homes. Following project completion, management will migrate to the Virtual Trials Archives, forming a legacy dataset which will be expanded to include international RCTs, and will be accessible to the wider research community for analyses.

**Discussion:**

Analysis of pooled IPD has the potential to inform and direct future practice, research and policy at low cost, enhancing the value of existing data and reducing research waste. We aim to create a permanent archive for care home trial data and welcome the contribution of emerging trial datasets.

## Administrative information

**Table:** 

Title [1]	Protocol for the development of a repository of individual participant data from randomised controlled trials conducted in adult care homes (Virtual International Care Home Trials Archive: VICHTA)
Trial registration {2a and 2b}.	N/A
Protocol version {3}	Version 5; 9 January 2021
Funding {4}	National Institute for Health Research: Health Service & Delivery Research (NIHR HS&DR); NIHR127234
Author details {5a}	Lisa Irvine; Jenni Burton; Myzoon Ali; Terry Quinn; Claire Goodman
Name and contact information for the trial sponsor {5b}	University of Hertfordshire Hatfield, Hertfordshire, AL10 9AB
Role of sponsor {5c}	The Sponsor played no part in study design; collection, management, analysis, and interpretation of data; writing of the report; or the decision to submit the report for publication

## Introduction

### Background and rationale {6a}

Approximately 418,000 people live in care homes in the UK, yet accessible, reliable data on care homes, their residents and staff are lacking. The dearth of accessible, high-quality data has been highlighted previously, but was starkly exposed in the recent and continuing COVID-19 pandemic [2]. Information about care home capacity, staffing, health and social care needs and resident demographics are each required in order to inform resource allocation and meet their care needs. Administrative data (e.g. UK Office of National Statistics census) provides information about age, sex and demographic change in care home population over time, but cannot be readily linked to the long-term health, function or quality of life of individual residents. Length of stay, life expectancy and mortality of the care home population are not reliably known. Large cohort studies of older adults give much richer health data, but the proportion of care home residents in such studies is low [3, 4]. For example, Cognitive Function and Ageing Studies (CFAS) reports on 543 residents and English Longitudinal Study of Ageing (ELSA) reports on 303 residents [5, 6]. Internationally, large care home datasets are available, for example, through insurance schemes in private healthcare systems. However, with any routinely collected data, there are concerns over data quality, and for many of these registers, the data collected speak to a certain purpose only and may not contain the most relevant clinical information. In addition to problems sourcing data about residents, it is also difficult to find consistent information about the fragmented care home market, including staffing (ratios and retention), case mix, funding mix and ownership. The lack of publicly available national data on the care home sector is detrimental to those who live and work there. By failing to quantify the needs of those requiring care and their journey before entering care homes, local and national planning for the care needs of the ageing population living with dementia, multimorbidity and frailty is impaired [7]. For example, it is estimated that care home capacity will need to expand to facilitate care for those with complex needs to receive care at the end of their lives [8, 9]. However, current staffing, funding source, resident pathways to care and capacity to provide care are unknown.

Large randomised controlled trials (RCTs) conducted solely in care homes are a growing resource [10], collecting detailed information about every care home and resident they recruit. Whilst these RCTs may focus on a variety of health/care topics (e.g. falls risk, medication management, nutrition or infection) from the study team’s experience of working with various care home trials, we know that there is much overlap in outcome measurement and information collected on both residents and the care home structure. Trials in care homes monitor participants regularly, often for up to 1 year. Outcome measures, health resource use and clinical events as well as care home characteristics can therefore be tracked over this period, allowing for longitudinal analysis. Secondary analysis of individual participant data (IPD) allows for more complex and flexible analyses than is possible with only summary-level results. Whilst single care home trial datasets are valuable, if IPD from existing trials could be *pooled*, they would collectively provide a much larger, richer dataset on residents and staff of care homes. Repurposing care home trial data would permit rapid synthesis of large IPD through which to generate evidence based on high-quality data. This principle aligns with current moves towards improving efficiency and reducing research waste [11], a theme of increasing importance to funders and peer reviewers. Pooled IPD would permit exploratory analysis to better understand the care home population, reduce duplication of effort and refine and pilot future research questions. The International Committee of Medical Journal Editors has reiterated its commitment to improve trial transparency by sharing IPD from RCTs and registries [12] and strive to normalise the sharing of de-identified trial data [13]. Clinical trials units have also signalled their support [14], and all trials started after January 1, 2019, must include an IPD sharing plan in their trial registration [13].

#### Data repository models

**Fig. 1:**
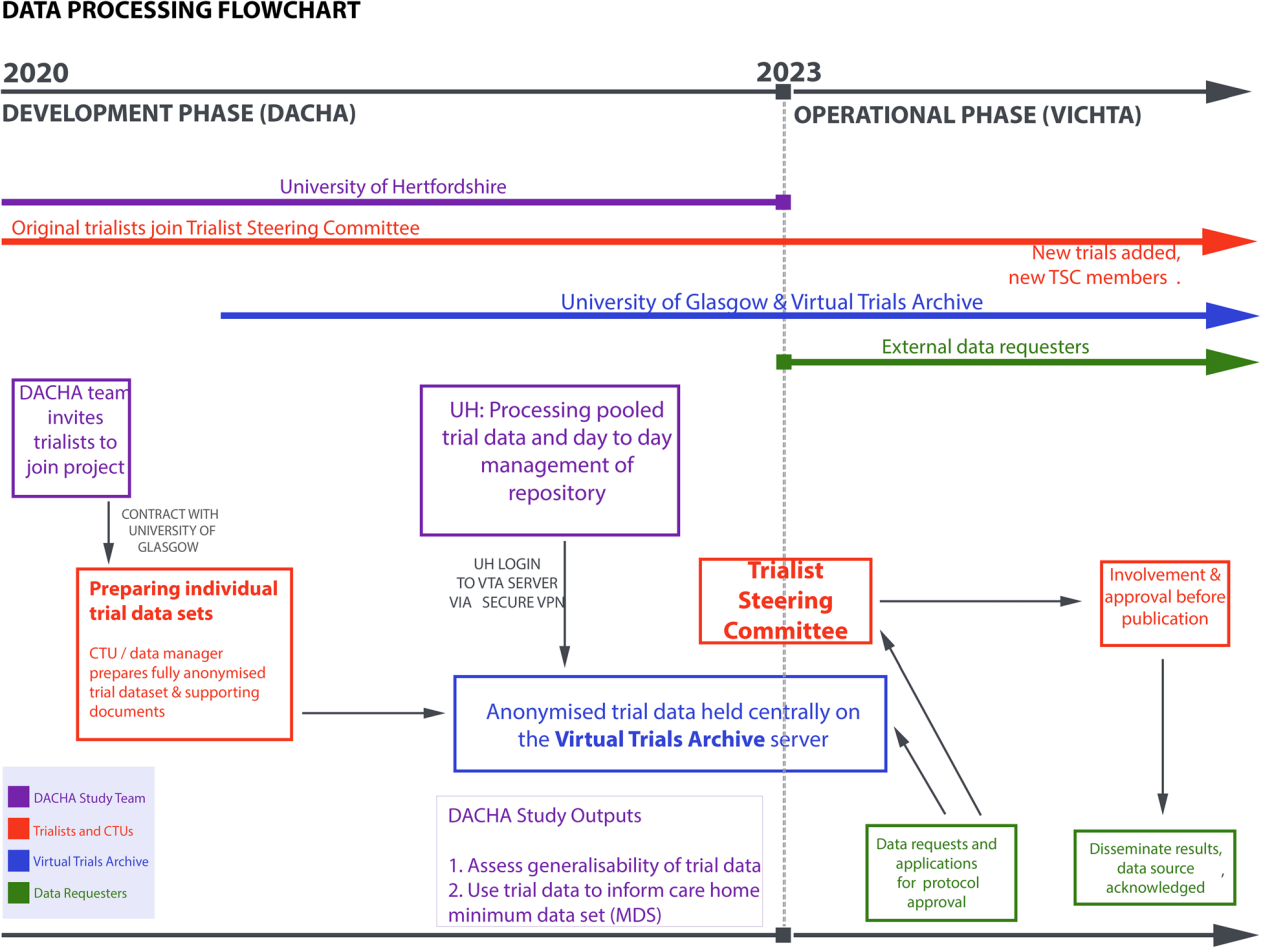
Data processing flowchart

Clinical data repositories such as Clinical Study Data Request (CSDR), Project Data Sphere and Yale University Open Data Access (YODA) Project are available to access IPD from single trials [15]. To allow data from *multiple* trials to be pooled into a single source within a secure data infrastructure, we will replicate the model developed by the Virtual Trials Archives (VTA) [16]. VTA was established in 2001, bringing together multiple, large, international data sets from completed clinical trials on stroke research [17, 18]. It has since expanded to include two additional repositories in areas of cardiovascular and cognition (VICCTA) and renal transplantation (VIRTTA) [19]. VTA is a not-for-profit collaboration, with datasets hosted by the Robertson Centre for Biostatistics (RCB) at the University of Glasgow, UK. The VTA facilitates a wide range of empirical and methodological research including recent projects on test accuracy [20], psychometrics [21], prognosis [22] and trial design [23]. Unlike with a traditional IPD meta-analysis [24, 25], a key tenet is that data should be used for novel research and not to test original hypotheses from contributed RCTs, though IPD meta-analyses are possible with permission of contributing trialists. Investigators can access data by submitting a research proposal on the VTA website. Following approval by the relevant repository Steering Committee (a virtual collaboration of the original trialists), data extraction is tailored to the specific research question, and the requesting investigator is granted access to analyse the bespoke data extract on a secure, online analysis platform, adhering to data security standard operating procedures. On completion, the anonymised data extract is archived centrally. The VTA is funded by administrative charges per data request, which supports data curation, storage, continued development and day-to-day administration of the resource. VTA has a well-established governance infrastructure, with the ability to host data securely on a working data-sharing platform, and expertise to manage future trial inclusion and data access requests. To enable the care home trial repository to operate on a long-term basis, we are working closely with the VTA from the outset. Once operational, the repository will formally migrate to the VTA, where it will be named the Virtual International Care Homes Trials Archive (VICHTA; see Fig. 1 and Table 2 in Appendix 3).

This protocol describes the creation of a care home trial repository as part of a funded project (the Developing research resources And minimum data set for Care Homes’ Adoption and use (DACHA) study; hereby described as the ‘development stage’) and also outlines plans for operation of the VICHTA repository that will be accessible beyond the DACHA study (hereby described as the ‘operational stage’). Our aims are to create a repository of IPD from RCTs conducted in adult care homes and use the repository data to conduct analyses to inform a care home minimum dataset relevant to the UK context [26].

### Study objectives {7}

#### Development stage (DACHA study)

1. Create a repository of IPD from trials conducted in UK care homes since 2010
2. Set up a Trialist Steering Committee, who will oversee data sharing and remain gatekeepers of their own trial data
3. Compare the pooled IPD with point estimates from administrative sources to assess generalisability of RCT data
4. Identify key resident characteristics and outcomes from within the trial repository, which could inform a national minimum dataset for care homes

#### Operational stage (Virtual International Care Home Trials Archive (VICHTA))

1. Enable new trials to be added to the repository beyond the DACHA project duration, including those from non-UK settings
2. Make pooled IPD available to external researchers to allow future secondary analysis

## Study design {8}

There will be four phases:

- Phase 1: Identifying trials and establishing the Trialist Steering Committee (TSC)
- Phase 2: Creating the repository, preparing data and pooling individual trial datasets
- Phase 3: Analysis of pooled data to inform DACHA study objectives
- Phase 4: Preparing for migration to Virtual Trials Archive and operation as VICHTA

### Phase 1: Identifying trials and establishing the Trialist Steering Committee

To be included in the proposed repository, trials must meet the following eligibility criteria {10}:

- Examination of any intervention conducted exclusively in an adult care home setting
- Minimum dataset of 100 participants
- Completed since 2010
- Trial conducted in the UK {9}
- Documented entry criteria
- Documented participant consent or assent following Health Research Authority approved procedure
- Monitoring procedures exist to validate data

Internationally, there is significant heterogeneity in the terminology used in practice and research to describe the settings in which long-term care is delivered [27, 28]. We have used the term ‘care home’ to describe care facilities that provide 24-h care to their residents, including those with and without on-site registered nursing staff.

#### Identifying trials

A scoping review identified potential care home trials for inclusion. As part of preparatory work, we contacted a small number of trialists who had completed RCTs in UK care homes to date. Based on provisional agreement from five of these trialists, we anticipate the repository will initially combine trial data for over 4200 residents from 250 care homes across the UK. Through an ongoing scoping review, we have identified a further thirteen potential trials, representing an additional 6000 residents from approximately 500 care homes. We anticipate this will increase further as the project develops, including the recruitment of international studies in the legacy dataset. Additional trials will be identified through an ongoing Google Scholar alert, systematically through concurrent reviews (Prospero: CRD42020155923), by contacting all trialists listed in the NIHR ‘Advancing Care’ Themed Review [10] (44 studies featured), the CLAHRC National Work stream Report [29] (32 studies featured), and snowballing techniques utilising the DACHA project management team, study steering committees and their professional networks.

#### Approaching/inviting trialists to share their data

We have created a database to track potentially eligible trials, where we will record how IPD are requested, collected and managed, and log of all contact with trialists. We will write to original trialists explaining the purpose of the repository and how it will operate. A reminder email will be sent 2 weeks after the initial contact if the trialist has not responded. If the trialist declines or does not respond, we will log this dataset as unavailable. Following a positive response, we will set up a meeting (phone, Zoom or face to face depending on trialist preference) to outline the project in more detail. If a trialist agrees to participate, they will be asked to sign a data transfer agreement that covers the transfer, use and storage of their trial data (see Terms of Reference, Appendix 1).

#### Establishing Trialist Steering Committee (TSC)

Contributing trialists will make up the TSC to oversee sharing, combining and repurposing of the pooled trial data. Whilst day-to-day co-ordination will be led by the DACHA co-ordinator at University of Hertfordshire (LI) and latterly the Virtual Trials Archive (MA), the TSC will agree on Terms of Reference for the collaboration, including the approval process for data requests, and will have the ultimate responsibility for all decisions regarding strategy, confidentiality, scientific matters and determining publication policy. This system mirrors the VTA, to which the care home repository will ultimately migrate.

The main role of the TSC during the DACHA-funded phase will be to provide advice on trial-specific details to aid with the pooling of datasets and better understanding of original data. Key information will be drawn from the original trial protocol, funders report and standard study documentation such as case report form templates and statistical analysis plans, but if any issues are not dealt with from those sources, we will seek clarification from the original trial team.

### Phase 2: Creating repository, preparing data and pooling individual trial datasets

#### Contributing trial data to repository

Once an agreement has been made to contribute data, trial data managers (e.g. within clinical trials units (CTU)) will be engaged to prepare datasets. As standard practice with individual participant data sharing models [30], only *completely anonymised* data will be held in the repository, to minimise the risk of reidentification. We will request that all data received will be fully de-personalised (such as converting ‘date of birth’ to ‘age at randomisation’). Full instructions on de-identification and how to transfer securely will be provided if necessary.

Additional documents to support datasets will be requested, including the trial protocol and data dictionary. Optional supporting documents will include blank, annotated case report forms, statistical analysis plans, relevant published outputs or grey literature about the trial. We will request evidence of ethical approval and consent procedure (e.g. blank consent and/or assent forms).

#### Repository data storage

The Virtual Trials Archive team have developed a DACHA data contribution form [16] where trialists can record information about the trial and complete a data sharing agreement. Following this, the trial dataset and all accompanying files will be transferred in a zipped, password-protected folder to the University of Glasgow’s RCB, using the University of Glasgow’s File Transfer Protocol, where it will be held securely for the duration of the DACHA study and beyond. As it does for other VTA repositories, the RCB will act as an independent data host, providing common format and access mechanisms. All data will remain on their server and analysed through their secure analysis platform, in accordance with standard conventions for data sharing initiatives. During the development stage, access to the data will be restricted to the core team (LI, JB and MA), who have undergone necessary data protection and confidentiality training. At the end of the DACHA project, the VTA will act as custodians of the data under the terms of the data transfer agreement.

#### Data preparation and quality checks

When trial data are submitted to the repository, the DACHA co-ordinator (LI) at the University of Hertfordshire (UH) will access the server remotely via a secure virtual private network. A data checking analysis plan will be developed, outlining procedures and decision rules for data pooling, according to established principles [30]. We will query any anomalies, including checks for invalid, out-of-range or inconsistent items with the trialist (or their nominated study contact) to ensure that the data are represented accurately. Trials may use the same outcome measure but administer it differently. If a measure could be completed, e.g. face-to-face with a member of the research team, or as self-report or as proxy-response from care staff, we will ensure this data are coded in a standardised way. Decisions on the standardisation will be made by consensus decisions with the wider TSC or delegated groups, e.g. trial statisticians. Where possible, we will request all individual domain scores for outcome measures as opposed to the single, composite scores. All trial datasets will be cross-checked against their respective protocol and statistical analysis plan to confirm how each composite outcome was derived. If the scoring was modified, we will seek clarification from the respective trialists in the TSC for their advice and interpretation on whether the composite outcome data should be removed or amended to enable pooling with other trial datasets. We will record the number and timing of measurement points and ensure all time points are labelled consistently.

We anticipate there will be a strong opportunity for methodological research to look at groups of measures, e.g. cognitive assessments, to attempt mapping or potentially harmonising similar variables [31, 32]. We would encourage external researchers to look at this in the operational phase; however, in the development phase, we will not attempt to harmonise non-matched data.

We anticipate most RCTs with an economic evaluation component will use a variant of the Client Service Receipt Inventory (CSRI) [33] to record information on resource use and costs alongside the trial. We will request all health service use questionnaires used in the trials and look for differences which may potentially impact the findings. Due to differences in price years and interpretation of unit costs, we will focus on resource use (e.g. number of GP contacts) as opposed to costs (e.g. total cost of GP contacts over the follow-up period). We will request datasets to include missing values where possible and not the imputed values. In developing the repository, we will not perform any missing data imputation.

#### Database of trial summaries

We have collated aggregate data available in each trial (generated through protocol papers and funders reports) and will build on this database as new trials are published. A summary of available data will be published on the VTA website, allowing viewers to identify what outcome measures have been collected multiple times, how care home characteristics have been recorded and contextual aspects of each trial, e.g. sample size and follow-up points.

The repository will host trials with a range of clinical focus—it is therefore likely that some measures will be unique to single trials. However, a combination of several key outcome measures—e.g. Barthel, Mini-Mental State Examination (MMSE), European Quality of Life Scale (EQ 5D) and Quality of Life assessment in Dementia (DEMQoL) [34–37], is used in almost all RCTs conducted in care homes. Additionally, clinical indicators such as hospitalisations, falls and death rates are routinely reported (see Table 1 in Appendix 2: examples of data available from each trial.)

### Phase 3: Analysis of pooled data to inform DACHA study objectives

When the initial set of trials has been added and variables prepared for pooling, we will temporarily lock the repository to allow two pre-specified analyses:

1. Identification of key resident characteristics and outcomes from within the trial repository, which could be used to inform the development of a minimum dataset (MDS) for care homes
2. Comparison of the pooled individual participant data with point estimates from administrative sources to assess the generalisability of RCT data

We will prepare a detailed research plan for each analysis, outlining the purpose of the request, objective/research question, plan for statistical analysis and repository variables requested. This research plan will then be circulated to the TSC for approval, as per future data requests from external analysts.

#### Informing development of a prototype minimum dataset (MDS) for care homes

Briefly, we will expand focus on what clinical, demographic and outcomes data from trials may be appropriate to include in a care homes MDS framework. We will categorise outcome measures into broad areas, e.g. cognition, anxiety and depression, pain, mobility, activities of daily living (ADLs) and specific clinical measures, and will focus on pre-specified outcome measures, in part identified through existing work on evidence reviews (PROSPERO CRD42020155923 and CRD42020171323). This identification and critique of relevant outcome measures within existing trials will help inform the development of a prototype MDS [26]. We will develop a quality assessment criterion to assess proposed outcome measures in terms of the following:

- What has been measured—baseline, processes of care, outcomes
- How data were collected (resident notes, researcher observation/assessment, use of routine data sources)
- Completeness of the data and where data are incomplete, what is the nature of this (i.e. death, unavailable, withdrawn consent, unable to complete, unclear)
- Where outcomes are measured across multiple studies, what are the range of values
- Where outcomes are measured over time, what is their sensitivity to detect change
- What information may be derived from collected data, e.g. comorbidity scoring based on medication usage

#### Generalisability of trial data

Briefly, we will conduct an evidence synthesis of key care home demographic information, by collating data from administrative sources, e.g. UK Census, Care Quality Commission. We will report baseline characteristics about care homes and residents as derived from all pooled trial data, tabulated for each individual trial and the pooled dataset. We will then compare point estimates from administrative sources with point estimates from the pooled IPD trial data, to evaluate how generalisable the repository data are, compared to alternative data sources.

### Phase 4: Preparing migration to Virtual Trials Archive

The VICHTA repository will be a legacy output of the DACHA project—a valuable source of high-quality, anonymised IPD to inform the development of future research, testing of hypotheses and optimisation of study design issues. We took an early decision to store all trial data solely on the University of Glasgow secure server, where the VTA is also housed. This means the repository will already have a permanent ‘home’ when the DACHA study ends. Management of the repository will be transferred from the DACHA team at the University of Hertfordshire (LI, CG) to the VTA team at the University of Glasgow (principally the VTA co-ordinator, MA). The VTA will maintain and update the VICHTA repository, and manage requests to access its data, in conjunction with the existing TSC.

Following formal migration to the VTA, external researchers may apply for data extracts, by submitting a project proposal (for review and approval by the TSC) and agreeing to the predefined VTA data sharing terms and conditions (see Appendix 1). At the proposal stage, TSC members may declare an interest in joining the analysis team of a proposed project and take an active role, thereby meeting the ICMJE criteria for authorship. All completed analyses will be forwarded to the TSC before submission for presentation or publication for review (see the data processing flowchart). The TSC is acknowledged on all publications using ‘on behalf of VICHTA collaborators’ by-line. Active involvement from each TSC member is encouraged but not essential, as data request decisions will be made by a quorum (see Table 2 in Appendix 3: summary of development and operational phases).

## Oversight and monitoring

### Data protection considerations {27}

In sharing any form of IPD, protection of personal privacy must be upheld [38]. A key factor to achieve this is to ensure trial data must be fully anonymised before it is added to the repository, to minimise the risk of reidentification. Electronic data will be stored securely on University of Glasgow server and will not be transferred or copied to any other location. Any paper documentation linked to the study will be scanned and stored as electronic data in the DACHA Study OneDrive as well as within the RCB servers. The paper version will then be destroyed. Together with the Data Protection Officer at the University of Hertfordshire, we have completed a Data Protection Impact Assessment to cover the research period of the DACHA study.

### Research governance {5d}

The University of Hertfordshire is the sponsor for the study, and their Ethics Review Board has approved this methodology (HSK/SF/UH/04185 approved 18 June 2020). Virtual Trials Archive has overarching university ethical approval for all their repositories and will update this through the University of Glasgow to include VICHTA. VTA will ask for indefinite ethics approval, subject to regular but infrequent reports at the discretion of the REC, e.g. 5-yearly, to minimise the administrative burden on both sides.

### Data security

Access to data extracts is restricted to individuals who have been granted access by the TSC only. The RCB is certified for ISO 9001:20015 for its Quality Management System and to ISO/IEC 27001:2013 for its Information Security Management System. RCB is audited every 6 months by the British Standards in Industry (BSI) and is regularly audited by its sponsors and clients both prior to and during studies. RCB has extensive experience in managing data in the context of privacy and data protection legislation, including the Data Protection Act 2018 and EU *General Data Protection Regulation*. Extensive data security procedures are in place including firewall protection, virus detection, daily backups, routine transaction logging, restricted access and on-site and off-site fire-proof storage of backups.

### DACHA project management

The Virtual Trials Archives are coordinated on behalf of the steering committees by MA (a coordinator with more than 10 years of experience in running VTA). During the DACHA study, the TSC will be co-chaired by JB (co-investigator on DACHA) and TQ, also based at the University of Glasgow and experienced in chairing other VTA repositories. Chairmanship can be reassigned at the nomination of the TSC. The research team has extensive clinical trials experience and all members are familiar with handling confidential anonymised personal health data.

The DACHA project has an independent Steering Group which will oversee this work package and the wider aims of developing a minimum dataset for care homes. This committee meets twice per calendar year.

### PPIE and public consultation {31a}

Patient and public involvement and engagement (PPIE) for the DACHA study will be led by the University of East Anglia and our expert-by-experience co-applicant (a family carer). PPIE will be represented on the DACHA independent steering group by two carers with family living in care homes. A PPIE panel is planned to work as the hub of PPIE activities, made up of 8–10 people representing care home staff, managers, family carers of care home residents and representatives of people with dementia. This group will meet 4-monthly, initially virtually. The care home resident PPIE contribution will be supported through two groups based in Norfolk care homes.

In addition to the PPIE panel, DACHA will have four regional groups of Expert Consultation groups, meeting annually. During these meetings, we will explain what data will be available in the trial repository, and then ask members to identify research topics that may be important for further investigation. Residents, their relatives, care home workers and managers are better placed to prioritise research questions on a more practical level; therefore, this exercise will ensure the right issues are being addressed.

## Discussion

This protocol defines the methods to curate a repository of care home trials for IPD analysis. It uses the existing, established infrastructure of the Virtual Trials Archive [1] to create this resource for informing the DACHA study and generating a legacy repository which will be expanded to include international care home trial datasets for future researchers. This represents an efficient use of existing research resources, enhancing the value from existing data and reducing waste [39, 40]. We are aware of one IPD meta-analysis combining the US and Dutch nursing home data [41], but this is the first attempt to develop a care homes repository, to which new trials can continually be added. This model can and has been replicated across a range of health conditions, including stroke, atrial fibrillation, ischaemic heart disease, heart failure, diabetes and metabolic conditions, cognition, renal transplantation (http://www.virtualtrialsarchives.org/) and aphasia (https://www.aphasiatrials.org/aphasia-dataset/).

Those living in care homes are a vulnerable population, and research in this setting is challenging, not least due to high rates of incapacity and dementia [42]. Re-use of data is efficient, minimising burden and intrusion to residents and staff and reducing the need for primary data collection. It adds value to the original trial question—whilst most trials are framed as *health* research questions, IPD provides the opportunity to address the questions and priorities of *social care*, including experiences of living and dying in care homes. In the absence of standardised data sets about care home residents, trial data will help us to understand more about this under-researched population. Curating a resource which is based on the setting of care, rather than being disease-specific, is attractive as we recognise that many of the challenges posed by health and care services are in caring for those with complex multimorbidity. Furthermore, there are lessons to improve future trial design, by exploring the value of the assessments and measures used in care home trials, to understand their utility, feasibility and relevance to care home life. Many of these tools were designed for use in community-dwelling adults or those in hospital settings and their applicability to the population living in care homes has yet to be established. IPD analysis can help address these questions, which are otherwise unanswered.

## Study status

Protocol version 4.

The project began on January 6, 2020. It is funded via the DACHA Study (NIHR127234) until October 30, 2023, after which the repository (VICHTA) will be maintained by the Virtual Trials Archive. We anticipate pooled datasets will be available for sharing by late 2023.

## Data Availability

Data sharing is not applicable to this article as no datasets were generated or analysed during this early planning phase of the study. Pooled data will be made available at the end of the DACHA development phase. A standardised approval process must be followed to gain access, which will be managed by the Virtual Trials Archive.

## Appendix 1: Trialist Steering Committee: Terms of Reference

### The Virtual International Care Home Trials Archives (VICHTA) terms and conditions for Steering Committee Membership

The Virtual International Care Home Trials Archives (VICHTA) will be an international repository for anonymised data from completed clinical trials that were set in care homes. This will be a not-for-profit collaboration hosted by the Robertson Centre for Biostatistics and the University of Glasgow.

Data will primarily be sought for use in the ‘Developing research resources and minimum data set for Care Homes’ Adoption and use’ (DACHA) study and will be contributed to VICHTA upon completion of the project to form a legacy dataset that can be interrogated for novel exploratory analyses. As per existing regulations that govern the stroke and cardiovascular sister archives (VISTA and VICCTA, respectively), we will not permit the reanalysis of treatment effects or reanalysis of the aims of the original trials.

Trialists that contribute anonymised RCT data to VICHTA will form the Steering Committee (1 named representative per trial, as per existing regulations). The role of the Steering Committee will be to:

1. Provide any additional information about relevant trial’s data, as appropriate, to aid in data cleaning and compilation
2. Review proposals to use data from VICHTA for novel exploratory analyses
3. Provide constructive feedback and peer review of proposals and papers

The Steering Committee members may also declare an interest in joining the analysis team of a proposed project provided that they take active participation in the project and meet the criteria for authorship stated by relevant journals. All declarations of interest should be made at the proposal circulation stage, and not at the paper review stage.

All papers that are generated as a result of VICHTA analyses will carry the by-line ‘on behalf of the VICHTA Collaborators’, and a full list of the Steering Committee members will be included in the appendix of all manuscripts.

Whilst we understand that reviewing proposals, abstracts and manuscripts can take some time, we appreciate active involvement from each Steering Committee member. If, however, certain members are unable to actively review all outputs, decisions will be made by a quorum.

## Appendix 2

**Table 1:** Examples of data available from each trial

Type of data	Examples of data available
Trial level	Study design Duration of follow-up Timing of assessment points Intervention details Region/geographical area covered
Care home level	Staff ratios; staff retention Number of beds; bed occupancy rates Case mix; funding mix; ownership
Participant level	Inclusion and exclusion criteria for residents Age at randomisation Sex Ethnicity BMI Medical conditions, e.g. Y/N presence of dementia, diabetes, COPD, previous stroke Time living in care home Status at end of follow-up (alive/dead/lost to follow-up) Cause of death Health resource use during follow-up Hospitalisations during follow-up Medications Advanced care planning
Outcome measures	Individual domain levels Summary scores Resident-reported, carer-reported or researcher-reported responses Baseline measures Follow-up measures

## Appendix 3

**Table 2:** Development and operational phase differences

	DACHA (funded up to November 2023)	Virtual Trials Archive (December 2023 onward)
Name	DACHA Trials repository	Virtual International Care Homes Trials Archive (VICHTA)
Funding	HS&DR NIHR127234	Supported through data access fees
Analysis	• Internal to WP2 team only • Analysis on University of Glasgow (UG) server (VPN access for LI) • Focus on descriptive statistics	• Available to external researchers via for data request process • All analysis performed on VTA Analysis Platform • More advanced statistical analysis
Management	University of Hertfordshire (UH) and Virtual Trials Archive/Robertson Biostatistics Centre (UG)	Virtual Trials Archive/Robertson Biostatistics Centre (UG)
Inclusion criteria	• UK only • RCTs only • Older adult focus • At least 100 participants • Any intervention/condition • Published since 2010 • Evidence of resident/consultee consent	• Add non-UK studies
Publication policy	All TSC members full authors for the main paper describing the development of repository. For other publications arising: • By-line ‘on behalf of VICHTA collaborators’ • Option to get involved in research and full authorship dependent on ICMJE guidelines.	By-line ‘on behalf of VICHTA collaborators’ Option to get involved in research and full authorship dependent on ICMJE guidelines.
Analyses	• Generalisability of RCT data • Using the repository to inform Minimum Dataset	Open for external researchers
Independent Oversight	DACHA Steering Group	Robertson Biostatistics Centre, UG
PPIE	Led by Anne Killett and Priti Biswas at UEA	
Ethics	University of Hertfordshire HSK/SF/UH/04185 approved 18 June 2020	Amendment for VTA ethics via UG
Trialist Steering Committee (TSC) membership	Co-chairs: Jenni Burton and Terry Quinn Up to two representatives per trial—principle investigator; CTU delegate; statistician; ECR Minimal face-to-face contact; Zoom conferencing	Chair/co-chair may rotate over years Minimal face-to-face contact

## Abbreviations

CTU: Clinical trials unit
DACHA: Developing resource resources And minimum data set for Care Homes’ Adoption and use
IPD: Individual participant data
MDS: Minimum data set
PPIE: Patient and public involvement and engagement
RCB: Robertson Centre for Biostatistics
RCT: Randomised controlled trial
TSC: Trialist Steering Committee
VICHTA: Virtual International Care Homes Trials Archive
VTA: Virtual Trials Archive
